# Hazard Identification on a Single Cell Level Using a Laser Beam

**Published:** 2007-12-06

**Authors:** Xing-Zheng Wu, Tomohisa Kato, Yumiko Tsuji, Satoshi Terada

**Affiliations:** 1 Department of Materials Science and Engineering,; 2 Department of Applied Chemistry and Biotechnology, Graduate School of Engineering, University of Fukui, Bunkyo 3-9-1, Fukui-Shi 910-8507, Japan.

**Keywords:** optical beam deflection, single cell, toxic hazard, UV-visible light, H_2_O_2_

## Abstract

This research shows a novel method for hazard identification of a chemical and UV light on a single cell level with a laser probe beam. The laser probe beam was passed through interface of cell membrane/culture medium of a cultured human hepatoblastoma cell line HepG2. Deflection of the laser probe beam, which was induced by changes in concentration gradients due to the active materials movement across the cell membrane, was monitored. When a toxic hazard existed, a living cell was expected to be killed or injured, or cellular behaviors to be changed greatly. Then, the changing deflection signal from the living cell would become unchanged or altered in a different way. This was successfully demonstrated with cytotoxity of UV light and H_2_O_2_. Most of the cultured HepG2 cells showed changing deflection signals after 10 min illumination of UV-visible light longer than 370 nm, while almost all HepG2 cells showed unchanged deflection signal after 10 min illumination of UV-visible light with wavelength longer than 330 nm. The results suggested that UV light between 330–370 nm could kill the cells. Additions of H_2_O_2_ solution with different concentrations to the cell cultures caused the changing deflection signal from a living cell either unchanged or changed in different trend, suggesting toxicity of H_2_O_2_ to the cells. The results from the beam deflection detection agreed well with those obtained by the conventional trypane blue method.

## Introduction

There are huge numbers of synthesized chemicals, and the potential adverse health effects of human exposures to most of them have not been understood. Identification of whether a chemical is toxic hazard is usually done by human case reports, experimental laboratory animal studies, environmental research, and *in vitro* studies with cell lines.^1^–^3^ Among these methods, *in vitro* studies with cell lines are the fastest and easiest methods, where cell death or cell injuries or changes in cellular behaviour are examined in presence of a target chemical.^1^–^7^

The most conventional cell death or cell viability is determined by labelling method using a dye such as trypane blue^8^ or fluorescent dyes.^9^ Flow cytometry^10^ can provide living or dead information about individual cells, but usually requires fluorescent staining and cannot be used for direct monitoring of cells in the culture process. Various microscopic techniques such as fluorescence microscopy, which also requires labelling, have been widely used for observing cells. However, the microscopic techniques are labour-intensive tasks, prone to error and operator fatigue. Vibrational spectroscopies^11^–^14^ such as the Infrared and Raman spectroscopy are nondestructive and noninvasive methods for study of cells. However, infrared measurements cannot be carried out in cell cultures because of the strong absorption of water in the infrared spectral region.^11^–^12^ Raman signal from biological molecules is usually low and thus the application in cell study is limited.^13^–^14^

**Figure 1.**
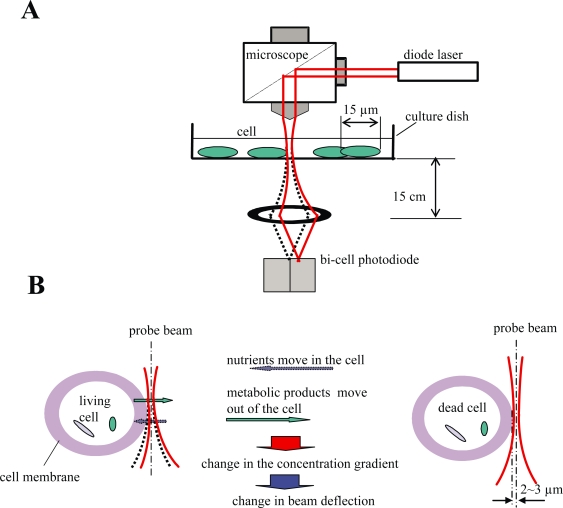
Illustration of the experimental setup for beam deflection detection (**A**) and its principle for diagnosing a single cell (**B**).

Very recently, we have applied the optical beam deflection (OBD)^15^–^18^ method to non-invasive diagnosing of a single living cell from a dead one.^19^ In this method, a laser probe beam is passed though an area of cell membrane of the cell as shown in Figure 1, and the deflection of the laser probe beam is monitored. When the cell is alive, movement of active materials across the cell membrane occur. For example, nutrients and oxygen move into, while metabolic products and CO_2_ move out of the cell, as shown in left of Figure 1-B. The movement of the materials induce changes in concentration gradients close to the cell membrane. The changes in the concentration gradient generate a change of the refractive index gradient of the medium near the cell membrane, which in turn generate a deflection of the laser probe beam. However, for a dead cell, little or no active material movements across the cell membrane occurs. Accordingly, little or no changes in deflection of the probe beam are generated as shown in right of Figure 1-B. In addition to the concentration gradients, a temperature gradient induced by heat from a chemical or biochemical reaction in cell activities also generates a deflection of the probe beam. However, the deflection caused by the temperature gradient is usually negligible compared to that induced by the concentration gradient, except for some special cases where a remarkable temperature difference is produced during the cell activities.^19^

The OBD diagnosis method for a single cell is based on monitoring of changes in concentration gradients in the vicinity of the cell membrane. In principle, this method can be used for determining any change in active movement of materials across the cell membrane of a single living cell. If a chemical is a toxic hazard to the living cell, the cell is expected to be killed or damaged; or cell activities are greatly affected by existence of the chemical. Then, the active movement of materials across the cell membrane is expected to cease or change greatly. This should be detected by monitoring of the deflection signal of the probe beam. In this work, we investigate the possibility of applying the OBD technique as a novel tool for fast toxic hazard identification of chemicals. Human hepatoblastoma cell line HepG2 was used as a model cell line. Cytotoxity of UV light and H_2_O_2_ on the cultured cells are investigated.

## The Experiment

Experimental set-up for the beam deflection was similar as that reported before.^19^ As shown in Figure 1-A, a culture dish with cells adhered on its bottom was placed on a platform of a microscope (CK40, Olympus of Japan). A laser beam from a diode laser (output power: 3 mW; wavelength: 667 nm) was introduced into the microscope. The probe beam was reflected inside the microscope, and then focused (spot size, about 3 ∼ 5 μm) to the cell membrane/culture medium interface of a cultured liver cell (about 15 μm in diameter) by an objective lens (N.A.: 0.1). An X-Y stage was used to adjust the distance between the laser beam and the cell membrane. The distance between the laser beam centre and the cell membrane was approximately 2 ∼ 3 μm. The probe beam exited from the culture dish and was focused to the centre of a bi-cell photodiode with a lens (focal length: 10 cm). The distance between the lens and the bottom of the dish was about 15 cm. The photo-currents i_1_ and i_2_ from the two sides of the bi-cell photodiode were inserted into an electric calculating circuit, where (i_1_ − i_2_)/(i_1_ + i_2_) was calculated and finally converted into electric voltage (volt) as outputs of the deflection signal.

The human hepatoblastoma cell line HepG2 was used throughout this work. This cell line expresses a variety of liver functions including albumin production. The medium used was Dulbecco’s Modified Eagle (DME) medium (Nissui, Tokyo, Japan) supplemented with 10% fetal bovine serum (FBS), 0.2% sodium bicarbonate, 10 mM N-2-Hydroxyethylpiperazine-N’-2-Ethane Sulfonic Acid (HEPES), 2 mM glutamine, and 0.06 mg ml^−1^ kanamycin. The cells were grown and adhered on the bottoms of culture dishes at 37 °C in humidified air containing CO_2_ at 5%.^20^ The viable and non-viable cell densities were conventionally determined by the trypan blue exclusion method using a Neubauer improved hemocytometer (Erma, Tokyo, Japan). Trypan blue was purchased from Dojindo (Kumamoto, Japan).

In experiments of light illumination, light from a high-pressure mercury lamp (Ushio Corp., Japan) was firstly passed through a water filter of 10 cm, then a cut-off filter with cut-off wavelength of 330, 350, or 370 nm, and finally illuminated to the culture containing 0.45 ml DME medium. The water filter was used for absorption of infrared light to avoid the temperature rise in the cell culture. The cut-off filter with cut-off wavelength of 330, 350, or 370 nm, cut off UV light with wavelength shorter than 330, 350, or 370 nm, i.e. it only permitted UV-visible light with wavelength longer than 330, 350, or 370 nm to illuminate the cells. The illumination period was 10 min. After the illumination, the culture dish was placed on the platform of the microscope, and its position was adjusted to let the laser probe beam pass through the interface of the cell membrane/culture medium of a cell. The deflection signal from the cell was monitored for about 500 s. Then, the position of the dish was readjusted for monitoring the deflection from another cell for 500 s too. This monitoring was sequentially repeated for 10 different cells. At last, cell viability of the illuminated cells in the culture dish was determined by the conventional trypan blue exclusion method.

In experiments of H_2_O_2_ hazard identification, the deflection signal from a cell in a culture dish containing 0.45 ml DME medium was firstly monitored for about 10 min. Then, 50 μl solution of H_2_O_2_ with certain concentration was added into the DME medium in the culture dish, and the deflection signal was continually monitored for another 10 min.

## Results and Discussions

**Figure 2.**
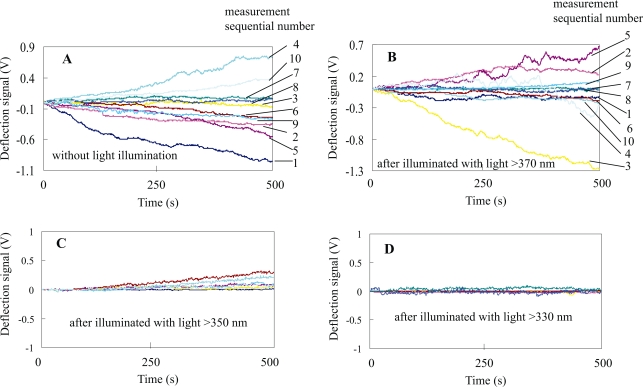
Deflection signals for different cells without light illumination (**A**) and after 10 min illumination of UV-visible light with wavelength longer than 370 nm (**B**), 350 nm (**C**), and 330 nm (**D**), respectively. In each of **A**, **B**, **C**, and **D**, the deflection signals were monitored sequentially from 10 different cells. Each plot in **A**, **B**, **C** and **D** represented the monitored deflection signal from a cell. The sequential No. corresponding to each monitoring was indicated on right of **A** and **B**, while the sequential No. was not indicated in **C** and **D**.

Because it is known that UV light could induce apoptosis relate to the cell cycle,^21^–^23^ the effect of the UV-visible light illumination to the cultured cells is firstly investigated here. Figure 2-A shows results of 10 sequential measurements of deflection signals from 10 different cells without light illumination, and Figures. 2-B, -C, and -D show those after 10 min illumination of different UV-visible light, respectively. The cells in Figures. 2-A, -B, -C, and -D are cultured at the same time and at the same conditions. Thus they have same cell viability before light illumination. In Figure. 2-A, deflection signal of sequential No. 3 changed little with time during the monitoring process. This suggested that the cell in sequential No. 3 was a dead one. Deflection signals in other sequential numbers changed with time although the change in sequential No. 7 and 8 were small. This suggested that they were alive.^19^ The different cells showed different deflection signals. This was because that the different cells had different active material movements, and the active material movements always changed with time.

In Figure 2-B, deflection signals in sequential No. 7 and 8 changed little with time, suggesting cells of the sequential No. 7 and 8 were dead. Others showed the changing deflection signal with time. This implies that most of the living cells were still alive after 10 min illumination of the UV-visible light with wavelength longer than 370 nm. On the other hand, less than half of the cells showed deflection signals changing with time in Figure 2-C (the sequential number was not indicated because the plots in Figure 2-C were too close or overlapped each other). This suggested that less than half of the cells were kept alive after 10 min illumination of UV-visible light with wavelength longer than 350 nm. Moreover, changes of the deflection signals with time in Figure 2-C were much smaller than those in Figures 2-A and 2-B, suggesting that the active materials movements of the living cells had changed greatly due to the light illumination with wavelength longer than 350 nm. In Figure 2-D, most of cells showed that the deflection signals changed little with time (the sequential number was not indicated too because of the overlap of the plots), suggesting that most of the them had died after the illumination of UV-visible light with wavelength longer than 330 nm. The results of Figure 2 suggest that UV light between 330 ∼ 370 nm has a strong killing effect on the liver cells.

The traditional trypane blue method was also used for determinations of the cell viabilities with and without the light illuminations. Table 1 shows the cell viabilities obtained by the trypan blue method. The cell viabilities did not change after the illumination of the UV-visible light with wavelength longer than 370 nm. This indicates that 10 min illumination of UV-visible light long than 370 nm would not kill the cells. On the other hand, the cell viabilities were decreased to 22 and 6% when the UV-visible light wavelengths were longer than 350 and 330 nm, respectively. This means that the 10 min illumination of the UV-visible light with wavelength longer than 350 and 330 nm could kill about 71 and 87% living cells, respectively, in comparison with the results without or with light illumination with wavelength longer than 370 nm. These results indicate that the UV light between 330 ∼ 370 nm has strong toxic effect to the living cells, and concurred with the conclusions obtained by the OBD method.

The method also can be used to real time monitoring of the UV illumination effect on a single living cell. The deflection detection system is constructed by introducing an UV illumination source directly into the microscope and the results will be reported later.

**Figure 3.**
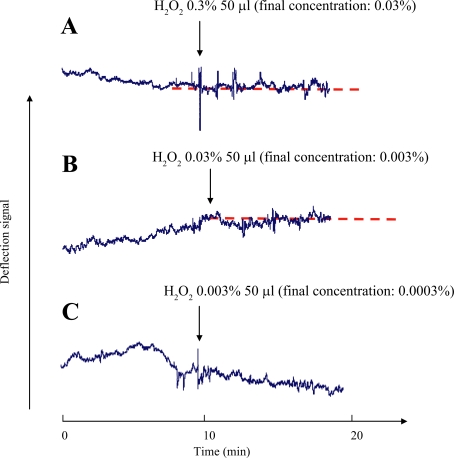
Effects of the additions of H_2_O_2_ solutions with different concentrations on the deflection signals from living cells. The arrows represent the time when the solutions of 50 μl H_2_O_2_ with different concentrations were added into 0.45 ml culture medium in the culture dish. The final concentrations of H_2_O_2_ in the culture medium were 0.03, 0.003, and 0.0003% in **A**, **B**, and **C**, respectively.

The method is further applied to assess toxicity of chemicals to the living liver cells. Hydrogen peroxide was chosen as a model chemical because it can induce apoptotic-like cell death,^24^ oxidative cell injury,^25^ and killing of cultured hepatocytes.^26^ Figure 3 shows changes of deflection signals of living cells before and after additions of different concentrations of H_2_O_2_ to cell cultures. In Figures 3-A, B, C, all of the deflection signals were changing with time before the addition of H_2_O_2_. This meant that the cells were alive before the addition of H_2_O_2_. However, after the addition of H_2_O_2_, the deflection signals were basically on the level of the dash lines as shown in Figures 3-A and 3-B although there are some noises. That is, the changing deflection signal remained unchanged. This implied that the living cells died soon after the concentration of H_2_O_2_ in the cell cultures increased from 0 to 0.03 and 0.003%. On the other hand, the deflection signal continued to change with time when the concentration of H_2_O_2_ was 0.0003% in Figure 3-C, but with different changing trend, compared with the results before the addition of H_2_O_2_. This suggested that although the cell was still alive, but it might have greatly damaged or injured after the concentration of H_2_O_2_ in the culture medium increased from 0 to 0.0003%. Figure 3 suggests that H_2_O_2_ is strongly toxic to the cultured cells and its toxicity increased with its concentration.

The living cell densities cultured in different concentrations of H_2_O_2_ were also determined by the trypane blue method. Table 2 shows that living cell densities decreased with the H_2_O_2_ concentrations. When the H_2_O_2_ concentration in the culture medium increased from 0 to 0.0003, 0.003, and 0.03%, the living cell density decreased from 2.27 to 1.46, 0.67, and 0.022 × 10^4^ cells/well, respectively. This also suggests that H_2_O_2_ is a toxic hazard to the cells, and its toxicity increased with its concentration.

Cell culture conditions are expected to affect the cell densities and cell activities greatly. Effects of the H_2_O_2_ and UV-visible light on beam deflection from living cells and the cell viabilities cultured under different culture conditions (for example, different CO_2_ concentrations rather than 5% used in this work) will be investigated in near future and results will be reported later.

As conclusion reported above, the conventional trypane blue method gave changes in cell viabilities or densities before and after treatment of a chemical or UV light illumination. Information on how many living cells had been killed by the treatment is able to be determined, however dynamic information related to the toxic effect of the chemical has not been established. The conventional method cannot be used for real-time monitoring of the toxic effect of a chemical on a single cell level. On the other hand, the OBD method is based on the real-time monitoring of change in concentration gradients near the cell membrane of a single living cell. The monitored OBD signal basically reflects dynamic change on the active material movements across the cell membrane before and after the treatment of the chemical. In addition to the real-time monitoring, cell viabilities also can be obtained by repeating the monitoring of the deflection signals from different cells, as reported in our previous paper.^19^ This method is expected to be particularly useful in probing changes of the material movements across the cell membrane in dying or damage processes induced by chemicals.

## Figures and Tables

**Table 1.** Effect of wavelength of UV-visible light illumination on living cell viability.

**UV-visible light wavelength (nm)**	**Power (W)**	**Cell viability (%)**
Without light illumination		93
>370	0.224	93
>350	0.251	22
>330	0.269	6

Illumination time: 10min

**Table 2.** Effect of the addition of H_2_O_2_ on living cell density.

H_2_O_2_ concentration (%)	0	9 × 10^−5^	3 × 10^−4^	9 × 10^−4^	3 × 10^−3^	9 × 10^−3^	3 × 10^−2^	9 × 10^−2^
Living cell density (× 10^4^ cells/well)	2.27	1.86	1.46	1.32	0.67	0.26	0.022	0
